# Treatment abandonment in children with Wilms tumor at a national referral hospital in Uganda

**DOI:** 10.1007/s00383-024-05744-7

**Published:** 2024-06-27

**Authors:** Sumayiya Nanteza, Ava Yap, Caroline Q. Stephens, Joyce Balagadde Kambagu, Phyllis Kisa, Nasser Kakembo, Geriga Fadil, Stella A. Nimanya, Innocent Okello, Rovine Naluyimbazi, Fiona Mbwali, Peter Kayima, Yasin Ssewanyana, David Grabski, Bindi Naik-Mathuria, Monica Langer, Doruk Ozgediz, John Sekabira

**Affiliations:** 1 Department of Pediatric Surgery, Mulago Hospital, Kampala, Uganda; 2 Center of Health Equity in Surgery and Anesthesia, University of California San Francisco, 550 16th St, 3rd Floor, San Francisco, CA 94158 USA; 3 Department of Hematology Oncology, Ugandan Cancer Institute, Kampala, Uganda; 4 Department of Pediatric Surgery, Makerere University College of Health and Sciences, Kampala, Uganda; 5 Department of Surgery, University of Virginia School of Medicine, Charlottesville, VA USA; 6 Department of Pediatric Surgery, The University of Texas Medical Branch at Galveston, Webster, TX USA; 7 Department of Pediatric Surgery, Ann & Robert H Lurie Children’s Hospital of Chicago, Chicago, IL USA

**Keywords:** Wilms tumor, Treatment abandonment, Pediatric oncology, Pediatric surgery, Low–middle income country, Global surgery

## Abstract

**Introduction:**

The incidence of pediatric Wilms’ tumor (WT) is high in Africa, though patients abandon treatment after initial diagnosis. We sought to identify factors associated with WT treatment abandonment in Uganda.

**Methods:**

A cohort study of patients < 18 years with WT in a Ugandan national referral hospital examined clinical and treatment outcomes data, comparing children whose families adhered to and abandoned treatment. Abandonment was defined as the inability to complete neoadjuvant chemotherapy and surgery for patients with unilateral WT and definitive chemotherapy for patients with bilateral WT. Patient factors were assessed via bivariate logistic regression.

**Results:**

137 WT patients were included from 2012 to 2017. The mean age was 3.9 years, 71% (*n* = 98) were stage III or higher. After diagnosis, 86% (*n* = 118) started neoadjuvant chemotherapy, 59% (*n* = 82) completed neoadjuvant therapy, and 55% (*n* = 75) adhered to treatment through surgery. Treatment abandonment was associated with poor chemotherapy response (odds ratio [OR] 4.70, 95% confidence interval [CI] 1.30–17.0) and tumor size > 25 cm (OR 2.67, 95% CI 1.05–6.81).

**Conclusions:**

Children with WT in Uganda frequently abandon care during neoadjuvant therapy, particularly those with large tumors with poor response. Further investigation into the factors that influence treatment abandonment and a deeper understanding of tumor biology are needed to improve treatment adherence of children with WT in Uganda.

## Introduction

Wilms’ tumor (WT), or nephroblastoma, is the most common pediatric renal tumor and the 4th most common childhood malignancy [1, 2]. However, significant global disparities exist in the outcome of WT, with evidence demonstrating survival rates > 90% in high-income countries (HICs), while those children in low- and middle-income countries (LMICs) have survival rates between 11% and 50% [3–7]. These disparities are likely compounded by the increased incidence in sub-Saharan Africa, delayed presentation, and more advanced disease [6]. In addition, even when children are finally diagnosed, many families abandon treatment, further exacerbating the poor outcomes in low-income countries.

In Uganda, children with oncologic diseases often experience delays in treatment that can be understood by the “three delays” model, which are due to delays in (1) the decision to seek treatment, (2) the ability to travel to the treatment facility, and 3) the receipt of adequate treatment once at the facility [8]. Treatment abandonment falls into the third delay and is defined as failure to begin or continue the planned treatment course after diagnosis, as well as any interruption of > 4 weeks in the scheduled treatment [9]. However, while the critical role that abandonment plays in treatment failure is well-recognized, the factors and phases related to treatment abandonment in low-income settings are poorly understood [10].

In some countries, such as Zambia, evidence has suggested that distance traveled and maternal education has a major impact on adherence to treatment plans [11]. While understanding treatment plans was important in Kenya, investigators there also found that financial barriers, inefficient services, and drug shortages also played an important role [12]. However, while in Uganda it is well-recognized that survival of WT is poor at 44%, little is known about the rates of treatment abandonment and factors that may contribute [13].

We sought to characterize the extent of treatment abandonment for children with WT in Uganda. The study goals were to (1) characterize the children with WT who abandoned treatment and (2) identify tumor-related factors associated with treatment abandonment.

## Methods

### Study design and setting

We conducted a combined retrospective and prospective cohort study following children < 18-years with WT diagnosed at a tertiary referral hospital in Uganda from January 2012 to April 2017. Retrospective chart review began in 2012 and prospective data collection in 2016. The institute manages patients with WT across Uganda and offers multimodal oncologic therapy, including chemotherapy, surgical resection, and at times radiation. All treatment plans are made to follow the international society of pediatric oncology (SIOP) guidelines and are formulated through multidisciplinary tumor board discussions. The standard treatment course includes neoadjuvant treatment prior to surgical resection [14]. Following the SIOP guidelines, the diagnosis was made using radiologic findings prior to the initiation of chemotherapy. Core biopsy was not routinely performed prior to surgery given the limited resource settings. Ethical approval was obtained through the Institutional Review Board of the Mulago National Referral Hospital’s associated medical school Makerere University of Health and Sciences. STROBE guidelines for cohort studies were followed [15].

### Participants

All patients with newly diagnosed and radiologically confirmed WT were included in the study. Patients with liver metastasis, pulmonary metastasis, and tumor thrombi were also included. Those patients with primary extra-renal WT and those who did not undergo neoadjuvant treatment at the study site were excluded. For those who were receiving treatment in 2016, participants were recruited prospectively, and written consent and assent, when applicable, were obtained. Those who were identified retrospectively (i.e., before 2016) had documentation of consent waived for chart review data collection. All children were followed until lost to follow-up or 6 weeks postoperatively, during which patients usually had a documented postoperative visit with oncology to determine the next steps of treatment. The 6-week mark was chosen as the cutoff as patients typically had their clinical status recorded up until this point. Longer follow-up was not possible due to a lack of a data registry and the difficulty of tracking long-term outcomes via chart review in the resource-constrained setting.

### Variables and outcomes

The primary outcome variable was the proportion of patients who abandoned treatment, defined as a patient with unilateral WT who did not complete neoadjuvant chemotherapy or surgery, or a patient with bilateral WT (stage V disease) who did not complete definitive chemotherapy. While surgical resection plus chemotherapy is the ideal treatment for bilateral WT, the study institution did not routinely offer bilateral nephrectomies for patients with bilateral WT given the associated postoperative morbidity. After surgical resection, these children would have been at high risk for end-stage kidney disease without ready access to renal dialysis or renal transplant at the study institution or within the country. Notably, a similar study in Malawi that included patients with bilateral WT reported 7 out of 8 deaths despite access to surgical resection, highlighting the lethality of this disease subtype [16]. Conversely, patients adhered to treatment if they had completed the above treatment pathways. Secondary outcomes included the percentage of patients who experienced short-term mortality, which was defined as a documented death, and completion of neoadjuvant chemotherapy treatment. Poor response was defined as interval tumor growth during neoadjuvant chemotherapy treatment. Medical chart review also collected clinical, histopathological, treatment modality, treatment response, and outcomes data. Treatment data included neoadjuvant chemotherapy and perioperative characteristics.

### Data sources

Following consent, participants’ caregivers completed a structured phone or in-person questionnaire to obtain details of the child’s demographics, pre-hospital symptomatology, treatment duration, and outcomes such as short-term mortality. Data were additionally obtained from chart review of outpatient appointments including chemotherapy treatment visits, inpatient admissions, and surgical encounters.

### Statistical analysis

Descriptive statistical analysis compared characteristics between patients who did and did not receive surgery. Chi-squared tests compared differences in categorical variables, while Student’s *t* test compared differences in continuous variables. Proportions of patients who started neoadjuvant treatment, completed chemotherapy, and underwent subsequent surgery were calculated. Bivariate and multivariate logistic regression analyses estimated the odds of treatment abandonment with select variables that were significantly different between the surgical and nonsurgical groups. All statistical analysis was performed using Stata (College Station, TX) version 16.0. A *p* value of < 0.05 was considered statistically significant.

## Results

### Patient and tumor characteristics at presentation

Of 137 children, the mean age was 3.9 years and 53% (*n* = 73) were female (Table 1). In total, 62 patients (45%) abandoned treatment while 75 patients (55%) did not.

**Table 1:** Baseline patient characteristics of WT patients, divided into those who abandoned and adhered to treatment

	Total	Abandoned treatment	Adhered to treatment	*p* value
	*N* = 137	*N* = 62	*N* = 75	
Sex				0.48
Female	73 (53.3%)	31 (50.0%)	42 (56.0%)	
Male	64 (46.7%)	31 (50.0%)	33 (44.0%)	
Age	3.9 (2.7)	4.2 (2.9)	3.6 (2.5)	0.15
B symptoms	76 (55.5%)	34 (54.8%)	42 (56.0%)	0.89
Abdominal swelling	89 (65.0%)	36 (58.1%)	53 (70.7%)	0.12
Abdominal mass	28 (20.4%)	11 (17.7%)	17 (22.7%)	0.48
Months of symptoms	3.8 (4.6)	4.1 (5.7)	3.6 (3.4)	0.58
Hematuria	15 (11.2%)	8 (13.1%)	7 (9.6%)	0.52
Systolic BP	118.4 (20.6)	116.2 (21.6)	119.9 (19.9)	0.38
Distance from referral (km)	106.1 (114.6)	110.6 (108.3)	102.2 (120.3)	0.67
Distance from home (km)	122.4 (117.7)	127.6 (111.7)	118.2 (123.0)	0.64
Tumor site				0.21
Bilateral	12 (9.0%)	8 (13.1%)	4 (5.5%)	
Left	63 (47.0%)	25 (41.0%)	38 (52.1%)	
Right	59 (44.0%)	28 (45.9%)	31 (42.5%)	
Diameter > 25 cm	23 (16.8%)	15 (24.2%)	8 (10.7%)	0.035
Radiologic risk				0.55
Low	1 (0.7%)	0 (0.0%)	1 (1.3%)	
Intermediate	79 (58.1%)	35 (57.4%)	44 (58.7%)	
High	30 (22.1%)	16 (26.2%)	14 (18.7%)	
Undefined	26 (19.1%)	10 (16.4%)	16 (21.3%)	
Tumor stage				0.055
1	7 (5.2%)	1 (1.7%)	6 (8.0%)	
2	29 (21.6%)	13 (22.0%)	16 (21.3%)	
3	47 (35.1%)	16 (27.1%)	31 (41.3%)	
4	40 (29.9%)	21 (35.6%)	19 (25.3%)	
5	11 (8.2%)	8 (13.6%)	3 (4.0%)	
Chest metastases	21 (15.6%)	8 (13.3%)	13 (17.3%)	0.52
Liver metastases	31 (23.1%)	18 (30.5%)	13 (17.3%)	0.073
Hemoglobin	9.4 (2.2)	9.0 (2.5)	9.7 (2.0)	0.077

**Fig. 1:**
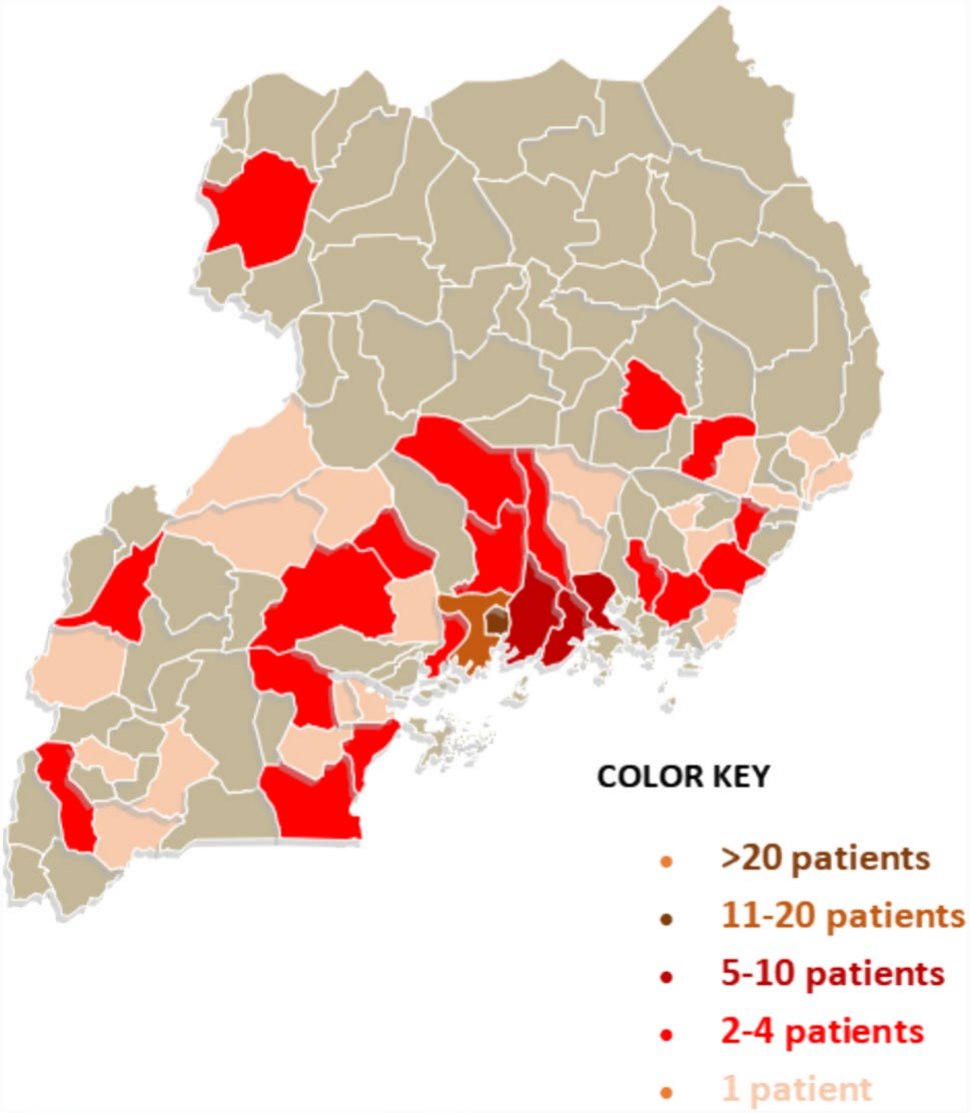
Geographical distribution of Wilms tumor patient referrals within Uganda

Families on average traveled 122 km (standard deviation [SD] 118) from home and 106 km (SD 114) from their referral center to reach the hospital, with no significant difference in distance traveled between those that abandoned and adhered to treatment. Most families came from the nearby districts of central, east, and west Uganda (Fig. 1). The most common symptoms were abdominal swelling (present in 65% or *n* = 89) and B-symptoms (present in 56% or *n* = 76). The mean duration of symptoms until the time of presentation was 3.8 months (SD 4.6).

At the time of presentation, 26.8% (*n* = 36) had early Stage I/II tumors, while 65.2% (*n* = 86) had later Stage III/IV tumors, and 9.0% (*n* = 12) had bilateral or Stage V tumors. High-risk presentation determined by imaging was present in 22% (*n* = 30) and metastasis to the chest and liver was present in 16% (*n* = 21). Compared to those that adhered to treatment, those that abandoned treatment were more likely to have tumors larger than 25 cm (24.2% vs 10.7%, *p* = 0.035).

### Treatment progression and abandonment

**Fig. 2:**
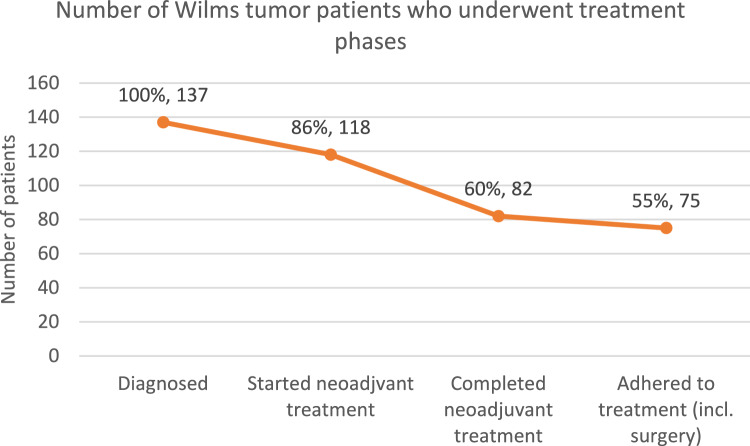
Proportion of patients in the cohort who underwent and completed treatment phases

Of those who were initially diagnosed with WT and seen at the oncology clinic, 86% (*n* = 118) were started on neoadjuvant chemotherapy, 60% (*n* = 82) completed neoadjuvant therapy, and 55% (*n* = 75) adhered to treatment through surgery or completion of definitive chemotherapy (Fig. 2). Of those who had surgery, the majority had total nephrectomies (99%, *n* = 70) and maintained an intact tumor capsule (94%, *n* = 68) (Table 2). Short-term known mortality was 18% overall (n = 25), 39% (*n* = 24) in the non-surgical group, and 1.3% (*n* = 1) in the surgical group.

**Table 2:** Treatment characteristics and outcomes of WT patients who abandoned or adhered to treatment

	Total	Abandoned treatment	Adhered to treatment	*p* value
	*N* = 137	*N* = 62	*N* = 75	
Time to neoadjuvant start	25.9 (60.3)	12.8 (13.8)	35.2 (76.8)	0.049
Neoadjuvant duration (days)	128.0 (82.5)	133.2 (77.5)	127.0 (84.0)	0.81
Neoadjuvant response				0.012
Good responder	50 (62%)	4 (31%)	46 (68%)	
Poor responder	31 (38%)	9 (69%)	22 (32%)	
Short-term mortality	25 (18.2%)	24 (38.7%)	1 (1.3%)	< 0.001

Compared to patients who adhered to treatment, those who abandoned treatment were more likely to wait longer to start chemotherapy (35 vs 13 days, *p* < 0.001) and respond poorly to chemotherapy (69% vs 22%, *p* = 0.012). (Table 2) In bivariate logistic regression analysis, abandoning treatment was associated with those who had a poor response to neoadjuvant chemotherapy (odds ratio [OR] 4.70, 95% confidence interval [CI] 1.30–17.0, *p* = 0.018) and tumors larger than 25 cm (OR 2.67, 95% CI 1.05–6.81, *p* = 0.039). Compared to patients with stage I tumors, patients with bilateral (stage V) tumors were associated with 12.0 times the odds of abandoning treatment (95% CI 1.05–137, p = 0.045). Time from diagnosis to starting chemotherapy was not significantly associated with a difference in the likelihood of treatment abandonment (OR 0.98, 95% CI 0.96–1.00, p = 0.097).

## Discussion

By following the SIOP protocol, survival rates for WT can be > 90%, especially for those with favorable histology or low-stage tumors (Tournade 2001). Treatment abandonment is therefore a source of preventable mortality and may contribute to the disparities in survival in LMICs. Our study was one of the first to report the proportion of surgical treatment abandonment in Uganda for patients with WT. Like other studies examining LMICs, we found that 45% of WT patients abandoned treatment in a tertiary hospital in Uganda [11, 12, 17–19]. In addition, our short-term mortality was 18.2% up to 6 weeks postoperatively, which did not account for long-term disease progression. Nevertheless, this mortality is still higher than in patients in HICs, which is consistently greater than 90% [3]. As a comparison, survival was reported in a systematic review of WT in Africa at 56.5% and a recent study from Southwestern Uganda demonstrated survival of 59% [19, 20].

In our study, the treatment phase when treatment abandonment occurred most frequently was during neoadjuvant chemotherapy treatment and was associated with poor chemotherapy response and large tumors. Given these findings, it is likely that through the treatment course, if the treatment does not appear to lead to clinical improvement, patients and families may lose confidence in the treatment plan and see surgery as futile. In addition, poor response to treatment and negative treatment side effects may also result in mistrust towards the medical system, causing families to fall back on traditional remedies. To support this reasoning, previous studies have suggested that response to treatment and surgical wait times are critically important for treatment adherence in WT patients [21]. Therefore, it is critical to set expectations early for families about the likely side effects of chemotherapy, the typical surgical course, and the anticipated timing of surgery once chemotherapy is complete. Another study in Kenya reported that 63% of parents caring for WT patients misunderstood treatment plans, which was a risk factor for treatment abandonment [12]. Especially given the prior evidence demonstrating a link between parental education and abandonment [21], it is equally important to supplement such explanations with educational materials that are easy to understand and follow [17].

Previous evidence has also suggested that financial limitations to pay for medical care, transport, and accommodation are common factors associated with treatment abandonment in Sub Sahara Africa [12, 18, 22]. However, in our study, no significant differences existed in the distance traveled for care. Thus, contrary to previous evidence, this suggests that transportation cost was not a substantial barrier to receiving care. Furthermore, as our center is located at a public hospital, costs of specific oncologic services were typically covered, though patients often pay out-of-pocket due to medication and supply shortages, and still have to pay for indirect expenses such as transportation and accommodation. Multiple previous studies have suggested that coverage of treatment, travel, and associated costs (accommodation, hospital food) can significantly decrease treatment abandonment [23, 24]. Interventions shown to decrease treatment abandonment in these settings include the Wilms Africa Phase I study, a multicenter clinical trial that implemented uniform treatment guidelines and funding to cover treatment, travel, and associated costs, which reduced treatment abandonment from 23 to 12% (*p* = 0.001) and improved survival from 52 to 69% (*p* = 0.002) [23]. Similar interventions to promote treatment adherence could potentially be instituted within the Ugandan healthcare system with a locally driven approach.

One factor that we found to be protective against treatment abandonment was patients presenting with a primary complaint of abdominal swelling (75% of all patients). Notably, only 34% of patients presented with complaints of a discrete mass in the abdomen, which is a more typical presentation in other settings and often incidentally found on routine clinical examination [25]. The lower frequency of this finding may be due to the intermittent contact that children have with the healthcare system, as regular well-child checks, which are often done in HIC, are not routine. As a result, those patients who present with abdominal swelling may have a parent who is more attuned to the health condition of their child. As a result, this group may have a heightened level of care-seeking in diagnosis and subsequent surgery. Thus, one method for improving treatment adherence may be forming support groups and community outreach programs to connect parents from similar communities who have completed treatment with those who have not to broaden support systems for WT treatment.

19% of children in our study had unfavorable radiological findings, which is more than twice the prevalence in HICs (~ 7.5%) [26]. In addition, advanced disease (stage III or higher) was present in 72.6% of our patients, which is much higher than the 58% reported throughout Africa [19]. While a combination of delays in diagnosis and referrals in parents seeking health care likely contribute to these disease actors, it is also well-known that WT has a higher incidence and histologic grade in Africa [19, 26]. However, there has been little investigation into studying the genetic and molecular pathways that lead to such aggressive disease. This is despite the significant growth of targeted therapies for the oncologic disease under investigation in HICs. This strategy of incorporating immunotherapies into oncologic treatments helps to both reduce the toxicity of treatment and improve treatment response [2]. Given the known differences in WT in LMIC and HIC, a necessary step in the improvement of WT outcomes in LMIC is the investigation into the genetic factors that predispose children in our setting to higher rates of WT. This would likely not only improve outcomes but with decreased toxicity, also improve adherence to therapy. Further investigation into the tumor biology of WT in our setting is sorely needed and would likely save lives in both LMICs and in HICs. In addition, a routine biopsy of WT to ascertain favorable versus unfavorable biopsy may be helpful to counsel families and patients on their treatment course, set realistic expectations, and reduce treatment abandonment. However, in the LMIC setting, tissue diagnosis is limited by the resources available to obtain biopsies in the pre-treatment period, so judicious selection of patients in need of biopsies may be warranted (e.g. for those who have larger tumors but with an indolent course, suggesting favorable histology and therefore prognosis).

The limitations of this study included its retrospective and observational nature, such that measurements of risk factors and outcomes were limited to pre-existing data and may be subjected to confounding and missingness. Specifically, the study did not delve into the social determinants of health of the patients being treated, and how the family’s income, education level, or social support could influence their levels of treatment adherence. In addition, the small number of patients in each subgroup also limited the statistical analysis and precluded multivariate regression modeling. Furthermore, the short-term follow-up period limited the determination of long-term survival for WT in our setting. We also did not have consistent access to tumor histology or surgical pathology, which prevented risk-stratification of treatment abandonment based on pathologic aggressiveness.

Notably, a prior qualitative study in Malawi of caregivers of WT patients explored reasons for treatment abandonment through semi-structured interviews and focus groups. In this study, caregivers were burdened with financial costs, inadequate social support, fear of treatment complications, uncertainty of treatment rationale, and a reluctance to ask health personnel questions, all of which may contribute to treatment abandonment [27]. Another qualitative study from southwestern Uganda demonstrated that financial constraints, false perceptions of curative treatment or incurability, preference for alternative treatments that may have been driven by chemotherapy side effects were reasons that caregivers decided to abandon oncologic treatment for their children [28]. These findings may help guide an informed approach to mitigating treatment abandonment by addressing the caregivers’ perceived barriers to care. Further investigation needs to be tailored to those children treated for WT in Kampala, as prior studies are generalized to all pediatric cancers.

Therefore, the next steps for addressing treatment abandonment for WT patients in the Ugandan setting include a qualitative assessment of factors associated with abandonment through direct outreach to those families who abandoned treatment. In addition, funding for a local oncologic registry to allow for patients to be followed for 5 years after completion of therapy to understand the long-term outcomes for WT in our setting. Once the reasons for treatment abandonment have been further elucidated, recruitment of patient care coordinators who monitor treatment adherence and directly reach out to families when treatment is abandoned may be of significant help.

## Conclusion

Children with WT in Uganda are often diagnosed at a late stage and those who abandon treatment do mostly during adjuvant chemotherapy, leading to low adherence to SIOP guidelines. Poor responders to chemotherapy and children with large abdominal tumors are particularly susceptible to treatment abandonment. High levels of treatment abandonment during the neoadjuvant phase could indicate mistrust towards the medical system for patients who do not improve with chemotherapy. More investigation to garner an improved understanding of tumor biology, patient socioeconomic reasons for abandoning treatment could help inform outreach efforts to improve treatment response and adherence.

## Data Availability

Open access to the database is not made publicly available to protect the individual privacy of the participants’ data. Data can be made available on a case-by-case basis with direct communication with the authors.
